# Molecular Weights of Polyethyleneimine-Dependent Physicochemical Tuning of Gold Nanoparticles and FRET-Based Turn-On Sensing of Polymyxin B

**DOI:** 10.3390/s24072169

**Published:** 2024-03-28

**Authors:** Atul Kumar Tiwari, Munesh Kumar Gupta, Ramovatar Meena, Prem C. Pandey, Roger J. Narayan

**Affiliations:** 1 Department of Chemistry, Indian Institute of Technology, Banaras Hindu University, Varanasi 221005, India; atulkumartiwari.rs.chy19@itbhu.ac.in; 2 Department of Microbiology, Institute of Medical Sciences, Banaras Hindu University, Varanasi 221005, India; muneshg.micro@bhu.ac.in; 3 School of Environmental Science, Jawaharlal Nehru University, New Delhi 110067, India; ramovatarmeena@mail.jnu.ac.in; 4 Joint Department of Biomedical Engineering, University of North Carolina, Chapel Hill, NC 27695, USA

**Keywords:** gold nanoparticles, fluorescence sensing, FRET, polyethyleneimines, polymyxin B

## Abstract

Environmental monitoring and the detection of antibiotic contaminants require expensive and time-consuming techniques. To overcome these challenges, gold nanoparticle-mediated fluorometric “turn-on” detection of Polymyxin B (PMB) in an aqueous medium was undertaken. The molecular weight of polyethyleneimine (PEI)-dependent physicochemical tuning of gold nanoparticles (PEI@AuNPs) was achieved and employed for the same. The three variable molecular weights of branched polyethyleneimine (MW 750, 60, and 1.3 kDa) molecules controlled the nano-geometry of the gold nanoparticles along with enhanced stabilization at room temperature. The synthesized gold nanoparticles were characterized through various advanced techniques. The results revealed that polyethyleneimine-stabilized gold nanoparticles (PEI@AuNP-1-3) were 4.5, 7.0, and 52.5 nm in size with spherical shapes, and the zeta potential values were 29.9, 22.5, and 16.6 mV, respectively. Accordingly, the PEI@AuNPs probes demonstrated high sensitivity and selectivity, with a linear relationship curve over a concentration range of 1–6 μM for polymyxin B. The limit of detection (LOD) was calculated as 8.5 nM. This is the first unique report of gold nanoparticle nano-geometry-dependent FRET-based turn-on detection of PMB in an aqueous medium. We believe that this approach would offer a complementary strategy for the development of a highly sophisticated and advanced sensing system for PMB and act as a template for the development of new nanomaterial-based engineered sensors for rapid antibiotic detection in environmental as well as biological samples.

## 1. Introduction

**Scheme 1:**
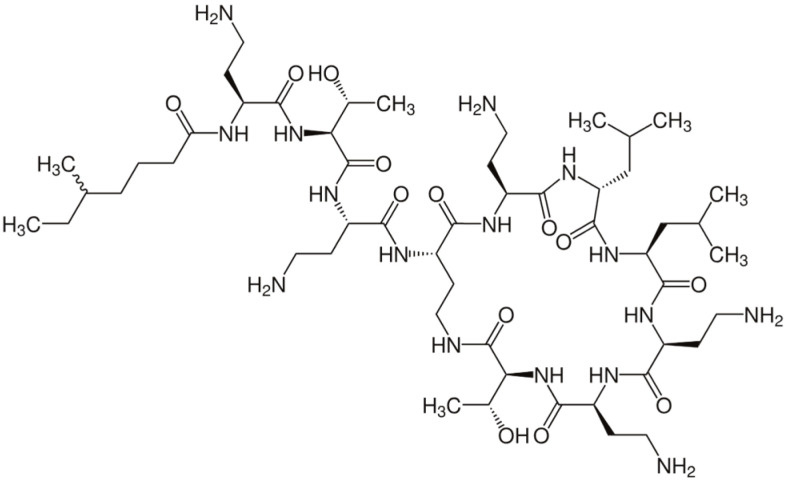
Structure of Polymyxin B.

Polymyxin B (PMB) is a polycationic lipopeptide antibiotic that is isolated from the bacterial species *Bacillus polymyxa* [1]. The primary structure of the molecule contains five primary amine groups, which make it a polycation at physiological pH (Scheme 1) [1]. PMB has several variants and is effective against Gram-negative bacterial infections. Due to the evolution of multidrug-resistance among bacteria, PMB has been contemplated for use as a prominent therapeutic agent [2]. It should be noted that inappropriate dosing of PMB could pose serious health complications such as nephrotoxicity, acute toxicity, and hyper pigmentation [3,4]. In addition, PMB has been added to feed for animals; since there are no regulations on PMB use, PMB is a common environmental contaminant [2]. The development of a rapid and economical detection approach is important for PMB monitoring of the antibiotic dosage in livestock products as well as environmental monitoring. The current methodologies reported for the detection of PMB include capillary electrophoresis [5,6], liquid chromatography–tandem mass spectrometry (HPLC) [7,8], thin-layer chromatography (TLC), multivariate calibration methods [9], and immunosorbent assay [10] approaches. Although these methods have some advantages, their drawbacks cannot be ignored, such as tedious sample preparation steps, time-consuming steps, high cost, use of an expensive apparatus, and use of expert manpower. Therefore, there is a pressing need to develop a rapid, sensitive, and economical approach for PMB detection.

Fluorescent probes have garnered significant attention because of their high sensitivity, high selectivity, low operating costs, simplicity, and rapid response. In recent decades, metal nanoparticles have attracted interest from researchers as an important material that has the potential to advance techniques for the detection and quantification of environmental contaminant traces in food, water, and soil, since they are associated with straightforward and economical implementation [11,12]. Among metal nanoparticles, AuNPs are an extensively utilized class of inorganic nanomaterials for a wide range of biomedical and catalytic applications due to their exceptional optical and electronic properties, including resonant coupling between the dipole of the excited molecules and the oscillatory electric dipole of the plasmonic electrons, which brings about an ultrafast energy transfer process [13,14,15,16]. Based on the above-mentioned unique nanoscale energy transfer behavior of AuNPs, a variety of efficient fluorescence biosensors have been created for the sensitive detection of proteins, enzymes, nucleic acids, and metal ions.

Recently, a semi-quantitative gold nanoparticle-based immunochromatographic paper strip was designed in which free PMB competes with the coated antigens to react with mAb-labeled AuNPs [17]. Using this strip, a visible detection limit of PMB in milk samples and animal feed samples was demonstrated up to 25 ng/mL and 500 μg/kg, respectively [17]. Similarly, Sonali et al. constructed a label-free modified immunosensor for the detection of polymyxin B in chicken eggs [18]. A conventional Au-SPE was fabricated with a specific colistin antibody using polyethyleneimine (PEI) modified by a carbodiimide-mediated amide coupling procedure; a limit of detection (LOD) up to 1.539 µg/kg in eggs with high specificity was achieved [18]. In addition to the immunosensor fabrication technologies, FRET-based “turn-on” detection of trace amounts of PMB was explored by using pairs that included rhodamine B (donor) and citrate-capped gold nanoparticles as acceptors [19]. The recovery (turn-on) sensitivity of the system was dependent on the relative concentration of AuNPs in a metal dye complex; an LOD for PMB up to 3.9 ppm was observed [19].

In this work, a simple, rapid, and one-pot synthetic procedure under ambient conditions for the reduction of gold nanoparticles was utilized for nano-geometry-controlled preparation of polyethyleneimine-stabilized gold nanoparticles, which was dependent on the molecular weight of PEIs (e.g., MW 750, 60, and 1.3 kDa. of bPEI). It should be noted that the role of the molecular weight of PEI in controlling the nano-geometry of silver nanoparticles has been reported by our group [20]. Accordingly, by taking advantage of FRET and the variable size of AuNPs employed, a selective and sensitive fluorescent sensor for the detection of PMB in an aqueous medium was developed.

## 2. Materials and Methods

### 2.1. Reagent and Chemicals

The materials and reagents used in this work were of analytical grade. All molecular weights of polyethyleneimines (50% *w*/*v* in H_2_O), chloroauric acid trihydrate (HAuCl_4_·3H_2_O), tyrosine, tryptophan, antibiotics, antifungal agents, glutathione, bovine serum albumin (BSA) fraction IV, and formaldehyde were acquired from Sigma Aldrich, Mumbai, India. Mercuric chloride, magnesium chloride, sodium chloride, calcium chloride, potassium chloride, ammonium chloride, nickel sulfate, sodium arsenite, and cobaltous were purchased from Merck, Bangalore, India. The other required glassware and plasticware were obtained from Tarsons, Mumbai, India. All of the experiments were conducted with ultra-purified HPLC-grade water.

### 2.2. Molecular Weight of Polyethyleneimine-Dependent Synthesis of Gold Nanoparticles

All of the PEI-functionalized gold nanoparticles (PEI@AuNP-1, -2, and -3) were synthesized as per the pre-optimized protocol with a slight modification [21]. In short, 200 μL (10 mM) of tetra chloroauric acid (HAuCl_4_·3H_2_O) was placed in a 2 mL glass vial, followed by the addition of an aqueous solution (30 μL, 2 mg/mL stock) of each molecular weight (750, 60, and 1.3 kDa.) of bPEI in separated vials. The reaction mixture was kept stirring with a magnetic stirrer for 5–10 min, which was followed by the addition of 30 μL of formaldehyde (37% *w*/*v*). Stirring was continued for the next 20–30 min under ambient conditions until maturation to yield the dark red-pink color of PEI@AuNPs.

### 2.3. Physical Characterization of as-Produced Gold Nanoparticles (PEI@AuNPs)

The physicochemical properties of as-produced PEI@AuNPs were evaluated using UV–Vis spectroscopy (Hitachi U-2900 spectrophotometer, Hitachi, Ibaraki, Japan). Transmission electron microscopy (TEM) was utilized to determine nanoparticle shape and size (TEM, TECHNAI G2 20 S TWIN, Field Electron and Ion Company, FEI, Hillsboro, OR, USA). The diameters of PEI@AuNPs were measured using ImageJ software (version 1.54b 8 January 2023, National Institutes of Health, Bethesda, MD, USA); a statistical graph was plotted on Origin 8.5 software (Northampton, MA, USA). The CIE-1931 chromaticity data were evaluated using an online server platform https://sciapps.sci-sim.com/CIE1931.html (accessed on 24 December 2023). Fluorescence emission (FL emission) spectroscopy was conducted using a Hitachi F7000 fluorescence spectrophotometer (Tokyo, Japan). X-ray diffraction data were recorded on a bench top Mini-Flex 600 (Rigaku, Tokyo, Japan). A Malvern Nano Zeta-Sizer instrument (Malvern, UK) was employed to obtain dynamic light scattering (DLS) and zeta potential data from the gold nanoparticles. In brief, each prepared PEI@AuNPs was diluted, and the optical density was adjusted to 0.1–0.3; the DLS and zeta potential data were subsequently recorded. The obtained data were represented as mean size and zeta potential.

### 2.4. Experimental Setup for Sensing of Polymyxin B

All of the experiments were performed in ultra-purified HPLC grade water under ambient conditions and, for an adjusted pH of 7.4. 10 μL of each produced PEI@AuNPs was added to an aqueous solution of varying concentrations of PMB (ranging from 1 to 6 μM) in a 1 cm quartz cuvette; fluorescence spectra were recorded with an instrumental setup at 5/10 slit width, a PTM voltage of 700 volts, and an excitation wavelength of 350 nm. Before selecting the optimum excitation wavelength, a maximum emission screening was performed. The experiments were repeated multiple times to ensure the accuracy and consistency of the applied methodology.

### 2.5. Time-Resolved Fluorescence Lifetime Analysis

Time-resolved fluorescence decay data were collected using a Wi-Tech alfa 3 RT instrument (Ulm, Germany); laser pulses of 405 nm were used for excitation. Emission data were collected (at 90° to the excitation beam) for both PEI@AuNPs and PMB-added PEI@AuNPs. The instrument response function (IRF) was obtained at 295 nm and was noted to be ≤200 ps (FWHM). For all of the fluorescence lifetime measurements, peak count data were obtained when the emission polarizer was oriented at the magic angle with respect to the excitation polarizer. The decay was deconvoluted with respect to the IRF data; it was analyzed to obtain the lifetime distribution data.

## 3. Result and Discussion

### 3.1. Synthesis and Characterization of as-Produced PEI-Stabilized Gold Nanoparticles

**Figure 1:**
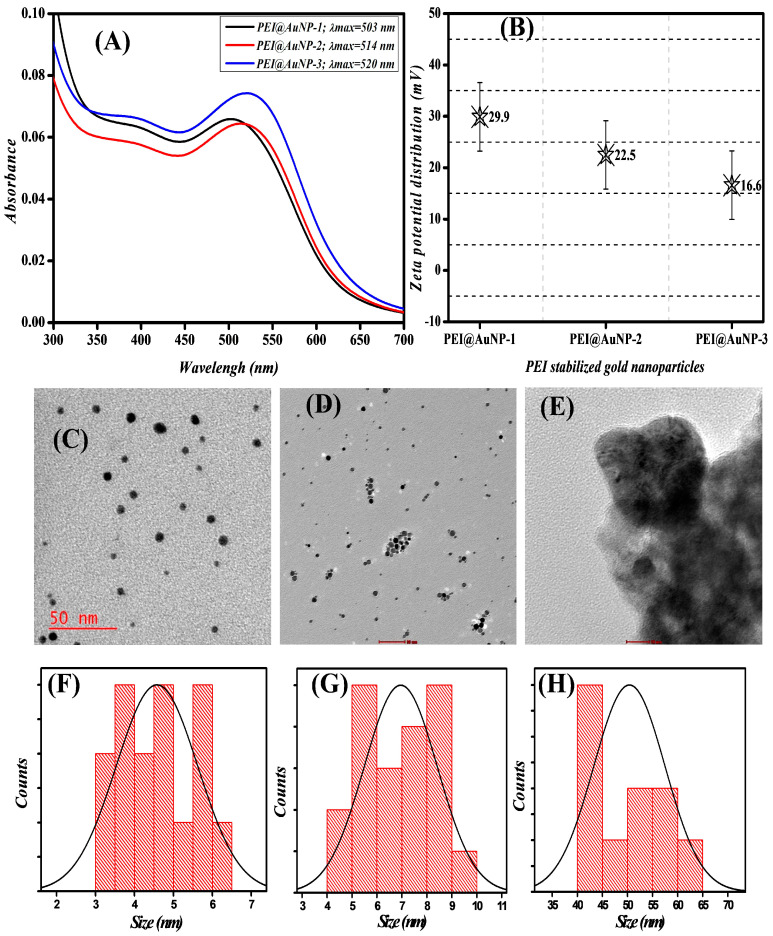
Physical properties of the as-synthesized gold nanoparticles. (**A**) UV–visible spectra of polyethyleneimine-functionalized AuNPs (PEI@AuN-1 to 3) and (**B**) corresponding zeta potential distribution. (**C**,**F**) TEM micrograph and actual size distribution plot of PEI@AuNP-1, (**D**,**G**) PEI@AuNP-2, and (**E**,**H**) PEI@AuNP-3, respectively.

Polyethyleneimine (PEI) is a versatile polymer used in many areas, from industrial applications to experimental gene transfection experiments [22]. Further, it has been used as a reducing and stabilizing agent for the conversion of gold and silver into nanoparticles; it is mostly used for surface modification of pre-prepared nanoparticles [20,21,23]. The role of PEI concentration in the synthesis of gold nanoparticles has been described for several purposes, from gene transfection to photo-thermal induction and glutathione sensing [21,22,23,24]. The effect of molecular weight on the nano-geometry of gold nanoparticles remains unevaluated. In this study, we explored the role of different molecular weights of PEI and their effect on the physico-chemical properties of gold nanoparticles, along with the interaction behavior for PMB under ambient conditions and neutral pH. The synthesis of PEI-stabilized AuNPs can take place in the absence of any organic reducing agent, in which PEI acts as a reducing agent in addition to a stabilizing agent [21,22]. However, the conversion kinetics could be slow and require an elevated temperature (at 60 °C) for 3–5 min. The presence of secondary organic reducing agents such as formaldehyde, THF/H_2_O_2_, and acetaldehyde enable the fast conversion of Au^3+^ to A^0^ at room temperature; the SPR properties of the product can be understood as a function of the organic reducing agents [21]. Herein, we used formaldehyde as a secondary reducing agent along with three different molecular weights of PEI (e.g., 750, 60, 1.3 kDa.) and an incubation time of 30–40 min for conversion and maturation of gold nanoparticles at room temperature. The resulting PEI-stabilized gold nanoparticles were denoted as PEI@AuNP-1 stabilized with 750 kDa. of PEI, PEI@AuNP-2 stabilized with 60 kDa. of PEI, and PEI@AuNP-3 stabilized with 1.3 kDa. of PEI, respectively. Figure 1A shows the UV–Vis absorbance spectrum of synthesized gold nanoparticles and demonstrates the effect of the molecular weight of PEI on the localized surface plasmonic resonance (LSPR) behavior of the nanoparticles. Accordingly, the high molecular weights of PEI (750 and 60 kDa.) represented localized surface plasmonic resonance (LSPR) bands around 503 and 514 nm, which were denoted as PEI@AuPEI-1 and -2, respectively; low-MW PEI (1.3 kDa.)-stabilized nanoparticles demonstrated an LSPR band at 520 nm (Figure 1A). This finding clearly shows that the molecular weight of polymer availability governs the physical properties of gold nanoparticle onset. Mechanistically, gold cations become trapped by the PEI branched chain structure, where amine groups start the reduction and nucleation of Au^3+^ [25]. During this process, large-molecular-weight PEIs produce AuNPs that are well dispersed due to strong repulsive force, which is mediated by a thick surrounding nest of PEI; this phenomenon provides a stable space until the completion of nucleation and prevents inter-particle interactions (e.g., agglomeration or aggregation). Similarly, if the PEI nest is smaller (low MW), the repulsive force may be insufficient to inhibit particle–particle interactions during reduction, which allows for a larger particle size and colloidal instabilities [25].

**Figure 2:**
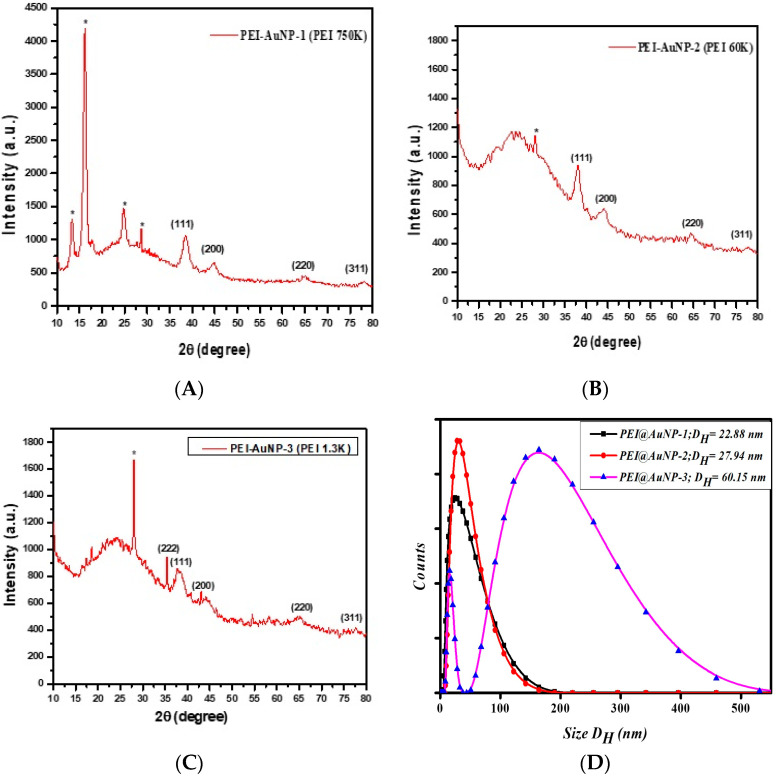
X-ray diffractograms of as-synthesized (**A**) PEI@AuNP-1, (**B**) PEI@AuNP-2, and (**C**) PEI@AuNP-3. (**D**) Hydrodynamic radii distribution plot of PEI@AuNP-1, -2, and -3. (*) mark showing unconfirmed peaks.

**Figure 3:**
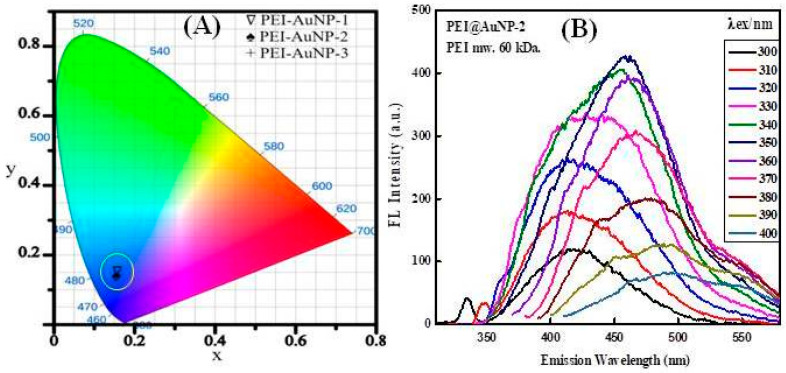
The fluorescence emission properties of synthesized PEI@AuNPs. (**A**) CIE-1931 chromaticity coordinates, and (**B**) excitation-dependent emission screening.

To confirm these results, we conducted zeta potential measurements, as indicated in Figure 1B. The presence of the charge density in PEI depends on the number of monomer and cationic amine groups; accordingly, PEI@AuNP-1, -2, and -3 demonstrated ζ potential distribution values of 29.9, 22.5, and 16.6 mV, respectively (Figure 1B). Interestingly, the shape and actual size of all three nanoparticles, PEI@AuNP-1, -2, and -3, followed the pattern of UV–Vis absorbance as indicated in Figure 1A; the PEI@AuNPs were ~4.5, 7.0, and 52.5 nm in size, with a spherical shape, respectively (Table 1 and Figure 1C–H). The TEM data for the nanoparticles justify the role of the molecular weight of PEI on the physical properties of the as-synthesized gold nanoparticles. Recently, a similar approach has been taken by our group for PEI-stabilized silver nanoparticles [20]; however, the role of the molecular weight in controlling the particle size was the opposite of that for the gold nanoparticles. Conclusively, a higher molecular weight of PEI induces the formation of small-sized gold nanoparticles; however, large-sized nanoparticles are observed in the case of silver nanoparticles. To investigate the role of PEIs on the lattice structure of the as-synthesized PEI@AuNPs, X-ray diffractometry was performed between 10° and 80° 2θ degrees and compared with JCPDS No 004-0784. Figure 2A–C attributed the characteristic peak appearances at 38.4°, 44.6°, 64.6°, and 77.7° for PEI@AuNP-1, -2, and -3 to hkl planes (111), (200), (220), and (311), respectively. These results indicated that Au was in the face-centered cubic (FCC) atomic arrangement. Further, the hydrodynamic radii data of PEI@AuNPs (Figure 2D) confirmed the size of nanoparticles. The fluorescence emission properties of PEI@AuNPs were determined by the CIE chromaticity coordinates (Figure 3A), which indicated that PEI@AuNPs emit blue color light when exposed to UV light (λ = 350 nm). Furthermore, an excitation-dependent emission pattern throughout the 300–400 nm range was observed, which was attributed to numerous trap surface states (Figure 3B). As demonstrated, with an increasing excitation from 300 to 350 nm, the FL emission intensity progressively rose without emission wavelength shifting. As the excitation progressed from 360 to 400 nm, the emission intensity progressively decreased; the results indicated that diverse trap surface states led to an excitation-dependent emission pattern for the as-made fluorescent PEI@AuNPs. The emission intensity was highest at an excitation wavelength of 350 nm; further experiments were conducted at the same excitation wavelength.

### 3.2. Effect of Polymyxin B (PMB) on the LSPR Properties of PEI@AuNPs

**Figure 4:**
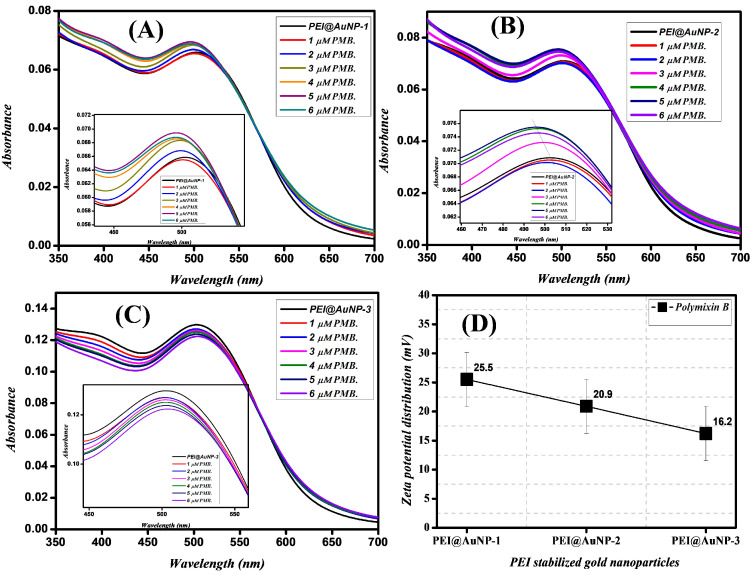
Absorbance spectra of PEI@AuNPs upon addition of different concentrations (1–6 µM) of PMB. (**A**) PEI@AuNP-1, (**B**) PEI@AuNP-2, and (**C**) PEI@AuNP-3. (**D**) Influence of PMB on the zeta potential of PEI-AuNPs.

The optical characteristics of metal nanoparticles (e.g., Ag, Au, and Cu) with a size below 20 nm are governed by the collective oscillation characteristics of the valence electrons [26,27,28]. These electrons have interactions with the electric field of the incident radiation; this phenomenon induces a dipole in the nanoparticle. The optical properties of metal nanoparticle dispersions are able to be quantified by evaluating the intensity of the absorption maximum due to SPR as determined from the UV–Vis spectrum [28]. To assess the effect of PMB on the SPR of PEI@AuNPs, we performed UV–Vis spectrophotometry of PEI@AuNPs in the presence of different concentrations (1–6 µM) of PMB as represented in Figure 4A–C. As noted in Figure 1A, the SPRs of synthesized PEI@AuNPs were 503, 514, and 520 nm, respectively. Interestingly, with increasing concentrations of PMB, the PEI@AuNP-1 and -2 demonstrated a significant blue shift in the SPR band along with intensity (as presented in Figure 4A,B inset). PEI@AuNP-3 was associated with a systematic reduction in the absorbance at 520 nm without any band shifting (as displayed in Figure 4C inset)). The results further indicate the role of PEI in considerable SPR shifting. Similarly, the zeta potential of PEI@AuNPs (Figure 4D) measured in the presence of PMB shows a decrease in PEI@AuNP-1 (29.9 to 25.5 mV) and -2 (22.5 to 20.9 mV), respectively (Figure 4D). However, no significant change in zeta potential was recorded in the case of PEI@AuNP-3 (as indicated in Figure 4D). Overall, PMB has a potential influence on the SPR and zeta potential of larger-molecular-weight PEI-stabilized gold nanoparticles (PEI@AuNP-1 and -2) as compared to low-molecular-weight PEI (PEI@AuNP-3). The fundamental cause of this phenomenon is still unknown and requires additional investigation.

### 3.3. PEI@AuNP-Mediated Sensing of Polymyxin B (PMB)

**Figure 5:**
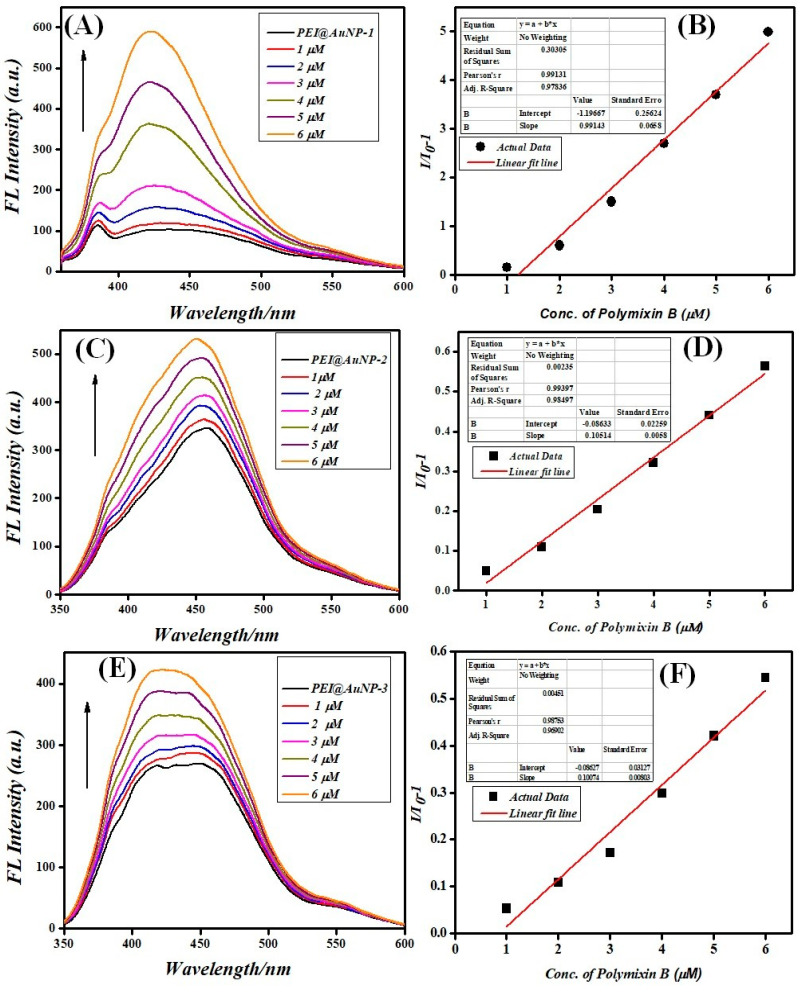
The fluorescence emission variation (excited at 350 nm) and kinetic parameters upon the addition of an increasing concentration (1–6 μM) of PMB in the PEI@AuNP suspension. (**A**,**B**) PEI@AuNP-1, (**C**,**D**) PEI@AuNP-2, and (**E**,**F**) PEI@AuNP-3.

Chemically, polymyxin B consists of two hydrophobic domains (e.g., the N-terminal fatty acyl and the D-Phe-L-Leu chain), which are separated by polar (Thr) and cationic (L-α-γ-diamino butyric acid) residues [29,30]. At physiological pH, the polymyxin B molecule exhibits structural amphipathicity [31]; however, the presence of PEI (basic condition) in the medium causes a negative charge on the amino residues of PMB to permit a selective interaction with the cationic PEI@AuNPs surface [32]. Therefore, the influence of the PMB concentration on the resulting fluorescence of PEI@AuNPs (the turn-on effect) was evaluated using the progressive addition of PMB (1–6 µM) into an aqueous solution containing a fixed concentration of each of the PEI@AuNPs. The fluorescence of the resulting system exhibits a dramatic change in the characteristic peak of emission (at 450 nm), with a progressive fluorescence turn-on (as shown in Figure 5). The total variation in the emission of PEI@AuNPs caused by the PMB was evaluated by calculating the ratio between the variations of emission (I/I0-1) through the modified Stern–Volmer equation as presented below [19]:

(1) $$\frac{I-I_{0}}{I_{0}}=K_{sv}[PMB]$$

*I*_0_ is the emission intensity in the absence of PMB, *I* is the emission intensity in the presence of PMB, *K_sv_* is the enhancement constant, and [*PMB*] is the concentration of polymyxin B.

As shown in Figure 5A,B, PEI@AuNP-1 demonstrated the highest variation in the *I/I0-1* ratio during the turn-on process over a linear range of PMB of 1–6 μM, with a Ksv of 8.35 × 10^5^ M^−1^ and R^2^ = 0.9783. PEI@AuNP-2 and -3 have a Ksv 9.4 × 10^4^ M^−1^ and 9 × 10^4^ M^−1^ along with R^2^ = 0.9849 and 0.9609, respectively, as indicated in Figure 5C–F. In addition to these parameters, the LOD represents a crucial parameter to evaluate the detection potential of a sensor to trace values of the analyte. As such, the LOD has been defined as the signal (S) to noise (N) ratio for a condition at S/N = 3; the LOD is 3.3 times the standard deviation of the y-intercept, which is divided by the slope of the regression [33,34,35]. Accordingly, the LOD of PEI@AuNP probes were calculated; the results demonstrated that PEI@AuNP-1 has the lowest LOD up to 8.5 × 10^−9^ M. In comparison, PEI@AuNP-2 and -3 exhibit values of 1.1 × 10^−8^ M and 1.1 × 10^−8^ M, respectively. These analytical parameters are better or favorably comparable to values previously reported in the literature as shown in Table 2. Hence, these results confirm that PEI@AuNP-1 with the higher slope and low LOD for polymyxin B justifies the ability of this straightforward experimental approach to be employed for the cost-effective detection of trace amounts of PMB in contaminant water samples.

### 3.4. Lifetime Decay Analysis

When a donor molecule is in close proximity (up to 10 nm or less) to an acceptor fluorescent probe, energy is transferred through non radiative dipole–dipole coupling, which causes an excitation of the acceptor probe [39]. The interpretation of time-resolved photoluminescence (TRPL) is highly useful due to its simplicity and conclusive results. The fluorescence enhancement properties of PEI@AuNPs (PEI@AuNP-1, -2, and -3) in the presence of PMB were recorded utilizing a double exponential function; the following equation was used [40]:

(2) $$D\left(t\right)=\sum_{i=1}^{n}ai\;exp(-\frac{t}{\tau i})$$

**Figure 6:**
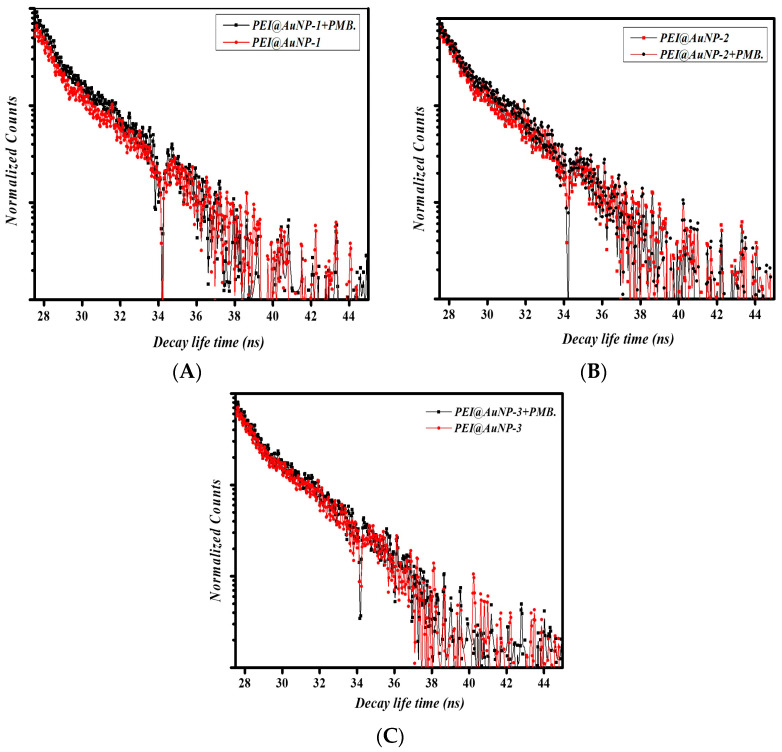
Time-resolved fluorescence lifetime of PEI@AuNPs in the presence of PMB. (**A**) represents PEI@AuNP-1, (**B**) represents PEI@AuNP-2, and (**C**) represents PEI@AuNP-3.

In this equation, *D*(*t*) represents the fluorescence decay time, τi represents the fluorescence lifetimes of numerous fluorescent forms, and (ai) represents the associated pre-exponential factors [39]. Accordingly, in the absence of a donor (PMB), the PEI@AuNPs demonstrated an average lifetime of 2.7 to 3.1 ns, respectively (Figure 6A–C), PEI@AuNP-1 shows a maximum decay time (3.1 ns) as compared to PEI@AuNP-2 and -3. Upon the addition of the highest concentration of PMB, the decay lifetime changed significantly. The results indicated an increase in lifetime to 3.9, 3.2, and 3.0 for PEI@AuNP-1, -2, and -3, respectively (Figure 6A–C). PMB acted as a donor (energy transfer due to the strong dipole–surface energy) for gold nanoparticles, which ultimately demonstrated fluorescence turn-on. It can be considered that PEI molecules acted as molecular spacers. It is important to understand the role of various molecular weights of PEI as spacers in order to consider the FRET process dominating during the electron transfer from PMB to PEI@AuNPs; these parameters are subject to further study.

### 3.5. Selectivity of PEI@AuNPs for PMB

**Figure 7:**
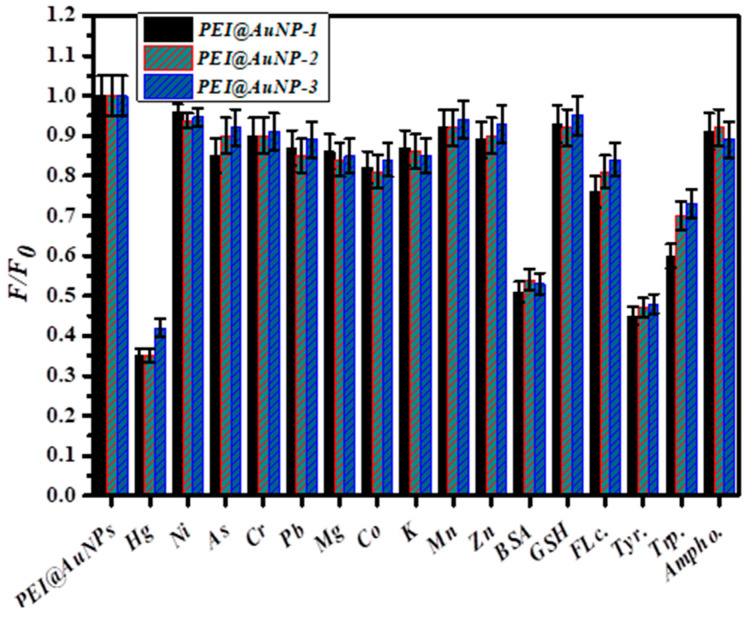
Selectivity of different PEI@AuNP probes against the various mono- and divalent cations along with biomolecule analytes, specifically BSA (bovine serum albumin), GSH (glutathione), FLc. (fluconazole), Tyr. (tyrosine), Trp. (tryptophan), and Ampho. (amphotericin B).

Selectivity is a crucial parameter in the development of a novel fluorescent detection probe. Accordingly, the PEI@AuNPs selectivity towards 100 μM of mono- and divalent cations (e.g., Hg^2+^, Ni^2+^, Cr^3+^, Pb^2+^, As^3+^, Mg^2+^, Co^2+^, K^+^, Zn^2+^, and Mn^2+^ ions), biomolecules (e.g., BSA, GSH, tryptophan, and tyrosine), and biocidal agents (e.g., fluconazole and amphotericin B) was evaluated. The results confirmed that the employed PEI@AuNP probes have excellent selectivity for PMB as indicated in Figure 7. However, Hg^2+^, BSA, tryptophan, and tryptophan have shown a significant level of fluorescence quenching activity against the probe. The Hg^2+^ binds with the nitrogen atoms of PEI, as suggested by a previous report by Kim et al. [41]. Further, the N 1s feature at 406.2 eV in Hg^2+^-PEI-AuNPs indicates the binding energy of the Hg^2+^-N bonds [42]. The binding of Hg^2+^ to the tertiary nitrogen of PEI@AuNPs ultimately quenched the fluorescence emission. On the other hand, the BSA protein consists of tryptophan (Trp), tyrosine (Tyr), and phenylalanine (Phe) residues; these residues absorb and emit ultraviolet light. The fluorescence intensity values of these residues decrease as follows: Trp > Tyr > Phe [43]. Further, each BSA molecule has two Trp residues; one of these residues is located at the hydrophobic pocket in domain II (Trp-212). The other residue is in domain I (Trp-134), which is on the surface of the molecule [44]. The emission energy value is very sensitive to the polarity of the environment due to the fact that the excited-state dipole moment of Trp is relatively large. Conformational changes in BSA can disturb the local environment near the Trp residues, which affects the fluorescence emission in the presence of gold nanoparticles. Similar dynamics can be used to understand the fluorescence quenching behavior of gold nanoparticles in the presence of Trp and Tyr residues. This phenomenon could be a significant limitation of PEI@AuNP probes for use in biological samples.

## 4. Conclusions

In conclusion, this study examined the potential role of various molecular weights of polyethyleneimines in controlling the nano-geometry of gold nanoparticles and subsequent FRET-based turn-on sensing of polymyxin B. The physico-chemical properties of the as-prepared gold nanoparticles were characterized through advanced techniques; the results justified the significant role of the molecular weight of PEI on the physical properties of gold nanoparticles, including the size, crystallinity, and zeta potential value. Interestingly, the higher molecular weight of PEI (750 kDa.) was associated with the formation of smaller nanoparticles with higher stability than lower PEIs (60 and 1.3 kDa.). Accordingly, FRET data involving polymyxin B and different PEI@AuNPs nanoprobes demonstrated considerable differences between the materials; PEI@AuNP-1 showed the lowest LOD (8.5 nM) for PMB. It is important to consider the suitability of PEIs as a spacer; the higher-molecular-weight PEI seems an excellent spacer molecule in this system; however, a more detailed study is required to establish this fact. In terms of selectivity, the PEI@AuNP probes have some limitations due to interaction with other biological molecules such as BSA and amino acids; nevertheless, the materials may have potential use for environmental monitoring of PMB.

## Figures and Tables

**Table 1:** The physical properties of as-synthesized PEI-stabilized gold nanoparticles.

Gold Nanoparticles	LSPR Band (nm)	Shape/Size (nm)	Zeta Potential (mV)	Hydrodynamic Radii (DLS) (d = nm)	hkl Planes
PEI@AuNP-1 (PEI; 750 k)	503	Spherical/4.5	29.9	22.55	(111), (200), (220), and (311)
PEI@AuNP-2 (PEI; 60 k)	514	Spherical/7.0	22.5	27.4	(111), (200), (220), and (311)
PEI@AuNP-3 (PEI; 1.3 k)	520	Spherical/52.5	16.6	60.15	(111), (200), (220), and (311)

**Table 2:** Comparison of different types of sensors for PMB. NA = Not applicable.

Probes	Method	Linear Range	Limit of Detection (LOD)	References
Thioglycolic acid-capped CdTe/CdS QDs	Fluorescence and RRS spectroscopy	0.09~5.25 µg·mL^−1^ and 0.02~6.0 µg·mL^−1^	27.5 ng mL^−1^ and 6.36 ng mL^−1^	[36]
Water-soluble perylene diimide (PDI) derivative	Fluorometric	1 to 2000 nmol/L	18.5 nM	[37]
Antibodies	ELISA-based method	5.0–192 ng/mL	30.6 and 1.8 ng/mL	[10]
Gold nanoparticle-based strip sensor	Indirect competitive enzyme-linked immunosorbent assay	0–100 ng/mL	13.13 ng/mL	[17]
Screen-printed gold electrodes (Au-SPEs)	Cyclic voltammetry (CV)	NA	1.539 µg/kg	[18]
Citrate-capped gold nanoparticle acceptors (Au NPs)	FRET	NA	2.82 µM	[19]
2, 7-Dichlorodihydrofluorescein diacetate (DCFH-DA)	Light-assisted noncompetitive immunoassay	8–20 μg/mL	70 ng/mL	[38]
Polyethyleneimine-stabilized gold nanoparticles (PEI@AuNPs)	Fluorescence (Turn-on)	1–6 μM	8.5 nM	Present work

## Data Availability

The original contributions presented in the study are included in the article; further inquiries can be directed to the corresponding authors.

## Abbreviations

PEI-AuNPs	polyethyleneimine-stabilized gold nanoparticles
LSPR	local surface plasmon resonance
SPR	surface plasmon resonance
DLS	dynamic light scattering
XRD	X-ray diffraction
PMB	polymyxin B
BSA	bovine serum albumin
LOD	limit of detection
FRET	fluorescence resonance energy transfer
PEIs	polyethyleneimines
TRPL	time-resolved photoluminescence.
